# The experiences and informational needs of women electing bariatric surgery: A qualitative content analysis of an online support forum

**DOI:** 10.1177/13591053251337218

**Published:** 2025-05-08

**Authors:** Jasmine Lester, Anna Chur-Hansen

**Affiliations:** University of Adelaide, Australia

**Keywords:** education, health care, obesity, social network, social support, surgery, weight loss, women’s health

## Abstract

This study aimed to explore how women leveraged online peer support to discuss their experiences and seek information related to bariatric surgery. Specifically, it sought to determine whether women are adequately prepared for the unique challenges associated with bariatric surgery and identify potential areas for improving preoperative education and support. An online search identified the *Bariatric Pal* online forum and sub-forum *The Gals Room*, of which 289 posts were selected and analysed using conventional qualitative content analysis. Five categories were developed: Connection with Forum Community; Life After Bariatric Surgery; Physical Symptoms and Experiences; Healthcare Concerns and Experiences; and Psychological Experiences. The findings highlight gaps in preoperative education for women, particularly regarding reproductive health and emphasise the value of online peer support in coping with and managing chronic health conditions. Further research is needed to inform improvements in preoperative and postoperative support for individuals undergoing bariatric surgery.

## Introduction

Obesity is a global public health challenge, carrying implications for the physical, psychological and social wellbeing of individuals. Obesity, characterised by excessive body fat, has reached epidemic levels with one-third of the global population living with overweight or obesity (Mohajan and Mohajan, 2023). Obesity is associated with various diseases, such as type 2 diabetes, cardiovascular problems and particular cancers (Mohajan and Mohajan, 2023). Bariatric surgery procedures involve the removal, or rerouting of the stomach and small intestine to facilitate weight loss, and is considered to be a highly-effective treatment for obesity (Cosentino et al., 2021). Common bariatric procedures include the Roux-en-Y Gastric Bypass, where a small pouch is created in the top part of the stomach, and is connected to the lower part of the small intestine, bypassing the remaining stomach and the first part of the small intestine, and the restrictive Vertical Sleeve Gastrectomy (gastric sleeve) (Lee and Dixon, 2017). The gastric sleeve involves removal of part of the stomach, with the remaining section of the stomach forming a sleeve-like structure with the small intestine (Ramada Faria et al., 2017). The gastric sleeve is considered the gold standard technique due to lower risks of complications and better weight loss outcomes (Ohta et al., 2022).

The benefits of bariatric surgery are well-documented, with surgery resulting in significant improvements or resolution in obesity-related diseases as well as reduced mortality and enhanced weight maintenance compared to other lifestyle or medicinal weight loss interventions (Hua et al., 2022; Noria et al., 2023; Pokorski and Głuch, 2022). Additionally, bariatric surgery can improve quality of life and psychological health, including reductions in anxiety, depression, and eating disorder symptoms as well as increased body image satisfaction (Fu et al., 2022; Law et al., 2023; Loh et al., 2021; Murton et al., 2023; Pyykkö et al., 2021).

However, despite the effectiveness and benefits of bariatric surgery, some individuals experience suboptimal results and adverse outcomes (Hua et al., 2022; Noria et al., 2023). For example, suboptimal weight loss and weight regain can occur after bariatric surgery due to maladaptive or unrestricted eating, nonadherence to dietary guidelines, and a sedentary lifestyle (Lauti et al., 2016; Noria et al., 2023). In addition, some individuals may continue to face psychosocial challenges, such as a potential worsening of anxiety or depression, eating disorders linked to restrictive diets, deterioration in body image, and changes in personal relationships (Cosentino et al., 2021; Coulman et al., 2017; Griauzde et al., 2018; Hua et al., 2022; Martens et al., 2021; Wright et al., 2022).

Bariatric surgery requires substantial psychological adaptation, including adherence to new lifestyle regimens, adjustment to an altered digestive system and coping with changes in weight and physical health (Coulman et al., 2017; Schlottmann et al., 2018). These adjustments can be overwhelming and distressing (Coulman et al., 2017; Martens et al., 2021; Schlottmann et al., 2018). Additionally, individuals who experience weight regain or suboptimal weight loss following surgery may face an elevated risk of psychological difficulties (Coulman et al., 2017).

Unrealistic preoperative expectations are commonly observed among candidates for bariatric surgery and may contribute to distress surrounding weight outcomes when post surgical outcomes fall short of expectations (Mercier et al., 2023). Misconceptions about expected amounts of weight loss and insufficient preparation for the necessary diet and exercise regimens are cited as contributing factors to these unrealistic expectations (Homer et al., 2016; Mercier et al., 2023).

It is recommended practice for candidates to undergo psychological evaluation prior to bariatric surgery, to assess mental readiness for physical and behavioural changes and to provide education about potential outcomes (Ackermann et al., 2020; Schlottmann et al., 2018). Despite this, research suggests that candidate reasons and expectations for surgery may not be thoroughly reviewed by healthcare professionals in the preparation stages (Groller et al., 2018; Homer et al., 2016; Marek and Heinberg, 2024; Mercier et al., 2023). Therefore, enhancing preoperative education could be an important component of managing unrealistic expectations surrounding bariatric surgery (Groller et al., 2018; Mercier et al., 2023).

Further, sex and gender are rarely considered in the prevention and clinical care of obesity (Cooper et al., 2021). It is well established that a gender disparity exists within bariatric surgery, with men less likely to undergo the surgery compared to women (Kochkodan et al., 2018). Some literature suggests that compared to women, men may experience challenges such as lower weight loss and increased complication rates (Kochkodan et al., 2018). Meanwhile, women are likely to experience unique challenges related to hormonal fluctuations, reproductive health, and body image (Cooper et al., 2021; Kochkodan et al., 2018). Given the gender disparities, the current state of preoperative education for bariatric surgery could incorporate information tailored to the gender and sex of the consumer (Fischer et al., 2014; Hult et al., 2022). This warrants an examination of cisgender women’s educational needs in the context of bariatric surgery. For example, the impact of surgery on women’s reproductive health is often overlooked. Conditions such as polycystic ovary syndrome (PCOS), anovulation, and menstrual irregularity are common in women with obesity due to hormonal imbalances, and these issues often improve after weight loss (Lee et al., 2020). However, it is unclear whether women are consistently informed of these potential changes prior to undergoing surgery. The potential for menstruation to resume after significant weight loss may indicate improved fertility and a need for contraception. Women living with obesity may already avoid hormonal contraception due to concerns about weight gain and literature suggests that contraception education is often inadequate for bariatric surgery candidates, particularly given that pregnancy is not advised following surgery (Mengesha et al., 2016; Riedinger et al., 2020). Hence, while bariatric surgery can impact women’s reproductive health and fertility, preoperative educational resources may not always address these issues.

These gaps in preoperative education may lead women to alternative information sources. For example, seeking online support provided by peers has been observed for a number of health conditions and is demonstrated to be effective in providing social support, yet still remains an underutilised method of sharing support and knowledge (Hossain et al., 2021). In general, individuals considering bariatric surgery may experience reductions in social support due to an existing stigma that depicts obesity as an individual weakness, further highlighting the benefits of online peer support networks (Ogle et al., 2015). When individuals feel misunderstood by the community or are ill-equipped with the knowledge provided by their healthcare professionals, they may turn to online communities for advice or support (Hossain et al., 2021; Ogle et al., 2015).

Misinformation within online forums (Farnood et al., 2021) is an issue and may be influenced by low health literacy or distrust in the mainstream healthcare system (Scherer et al., 2021). However, despite this, research shows that online health communication that focuses on sharing experiential insights to offer advice and social support to peers is valuable for people with chronic health conditions (Farnood et al., 2021; Hossain et al., 2021).

Online social support has been observed across many social media platforms related to bariatric surgery in both quantitative and qualitative research, including Facebook support groups and online forums (Athanasiadis et al., 2022; Koball et al., 2017; Robinson et al., 2020; Scarano Pereira et al., 2022; Willmer and Salzmann-Erikson, 2018). Within these online platforms, users often seek or provide support to one another, discuss physical and mental health challenges or accomplishments related to surgery, and engage in anecdotal discussions (Athanasiadis et al., 2022; Koball et al., 2017). Examining the experiences that women share with their online peers can help identify potential gaps in preoperative education or information, particularly regarding the effects of bariatric surgery on women’s health.

The present study used a qualitative methodology to address the following research aims:

1. Contribute knowledge regarding how and why women engaged in online peer support throughout the bariatric surgery process.
2. Explore the concerns and experiences that women expressed on an online forum dedicated to bariatric surgery.
3. Determine ways in which preoperative education can be improved to better support women undergoing bariatric surgery.

## Method

### Study design

Specific search terms were used to select an appropriate online discussion forum using Google, including ‘bariatric surgery’, ‘weight loss surgery’, ‘discussion board’ and ‘support forum’, yielding 6 potential discussion boards for selection. Three forums were excluded due to irrelevance to bariatric surgery. From the remaining three, one forum was excluded due to inactivity, and the second was excluded because it lacked a sub-forum dedicated to women. The remaining forum of choice was *Bariatric Pal*; one of the most globally active bariatric forums at the time of data collection (April, 2023). At this time, Bariatric Pal had 4,929,927 total posts, 414,756 topics and 419,464 members. There were 27 sub-forums within the Bariatric Pal forum, each housing different discussion topics specific to bariatric surgery (e.g. food and nutrition, success stories, fitness and exercise). To ensure the experiences specific to women were captured, the sub-forum *The Gals Room* was selected for textual data collection. This sub-forum included women who were planning or had undergone bariatric surgery, and included diverse discussion topics surrounding the physical, psychological and social aspects of bariatric surgery. At the time of data collection, the sub-forum constituted 38,763 posts dating back to 2003.

### Participants

The ethical considerations of collecting qualitative data from online forums include potential issues surrounding informed consent and participant privacy (Giles, 2017; Holtz et al., 2012; Smedley and Coulson, 2021). This study was granted low-risk ethics approval by the University of Adelaide Human Research Ethics Sub-committee (approval number: 23/19) with expectations of participant anonymity and public availability of data. Obtaining informed consent was not required nor possible. All participants were assigned a number, excluding all identifying information from the analysis for anonymity. Participants were members of the Bariatric Pal forum that posted in The Gals Room between January 2018 and March 2023. A 5-year time frame for data collection was selected to allow for women’s most recent accounts to be captured from a forum with substantial content, in field of surgery that is continuously developing (Ohta et al., 2022).

### Data collection

**Figure 1.**
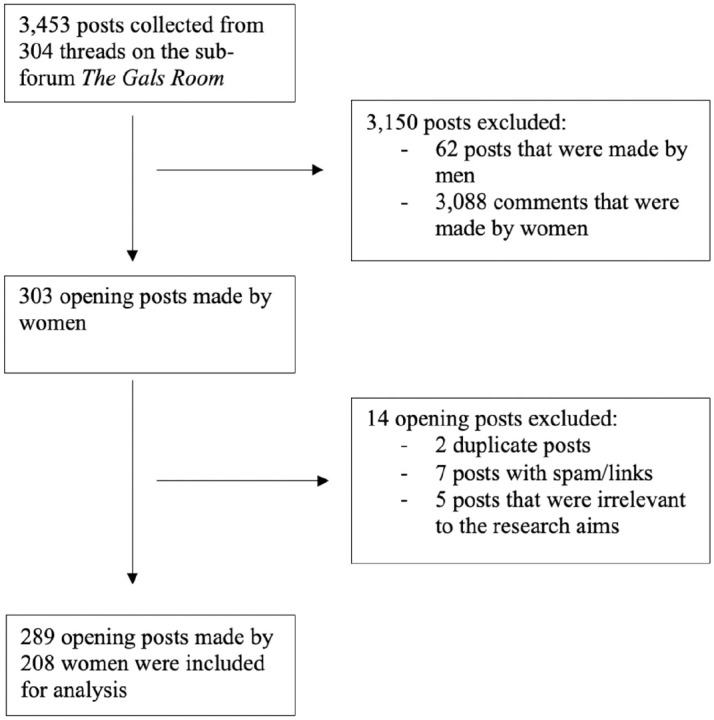
Forum post exclusion process.

The structure of online forums, as defined by Giles (2017), include multiple threads, consisting of an opening post and comments posted in response to this opening post. A post is defined as an individual comment in a thread (Giles, 2017). In this study, 304 threads were collected between January 2018 until March 2023. In total, 3543 posts were collected, including 304 opening posts, and 3149 comments in response to the opening posts. Only the opening posts were included in the analysis to provide an overview of participants’ experiences and concerns, highlighting how the online forum was used to engage with peers. All posts created by men were excluded. Figure 1 outlines the data exclusion process.

Each post was copied verbatim into Microsoft Excel, including the content of the post, post title, date of posting and poster username, presented chronologically. The data were deidentified and read repeatedly by the first author for immersion in the data. The resulting 289 posts were made by 208 women, indicating that multiple posts were made by the same participant. A list of participant demographics that were stated in each post was created.

### Data analysis

Conventional qualitative content analysis (Hsieh and Shannon, 2005), was used inductively; meaning that the codes, subcategories, and categories were created based on the surface and deeper level meanings contained in the data (Hsieh and Shannon, 2005). The analysis was guided by the following research question: ‘What experiences and concerns do women share with their peers on an online forum dedicated to bariatric surgery?’ The analysis aimed to condense extensive textual data into a summarised, categorical framework that captured key themes and experiences of women aligning with the research question and aims (Elo and Kyngäs, 2008). The software NVIVO 12 (version 12.7.0) was utilised to organise the data into subcodes, subcategories and overarching categories. For trustworthiness and rigour, the codes, subcategories, and categories were evaluated by the second author along with two other researchers.

In keeping with reflexivity (Jamie and Rathbone, 2022) and transparency, an audit trail was kept throughout the study, capturing the researchers’ decision-making processes and any personal reflections or assumptions (Renz et al., 2018). Neither author has had bariatric surgery: the second author is a Health Psychologist who works with men and women pre and post bariatric surgery. The Standards for Reporting Qualitative Research (SRQR) checklist was used to assess the methodological quality and reliability (see Supplemental File 1).

## Results

### Participant demographics

Of the 147 women that stated country of location, 93% were from the United States of America (USA). The type of surgery each participant received was recorded if stated, including participants in the preoperative phase (See Table 1).

**Table 1.** Type of surgery reported by participants.

Type of surgery	*n*	% of total
Vertical sleeve gastrectomy (gastric sleeve)	85	40.9
Preoperative phase	54	26.0
Roux-en-Y gastric bypass	35	16.8
Surgery type not defined	27	13.0
Biliopancreatic diversion with duodenal switch	4	1.9
Laparoscopic adjustable gastric banding (LAP-BAND)	2	1.0
Single anastomosis gastric bypass	1	0.4

*Note. N* = 208.

#### Categories, subcategories and subcodes

**Figure 2.**
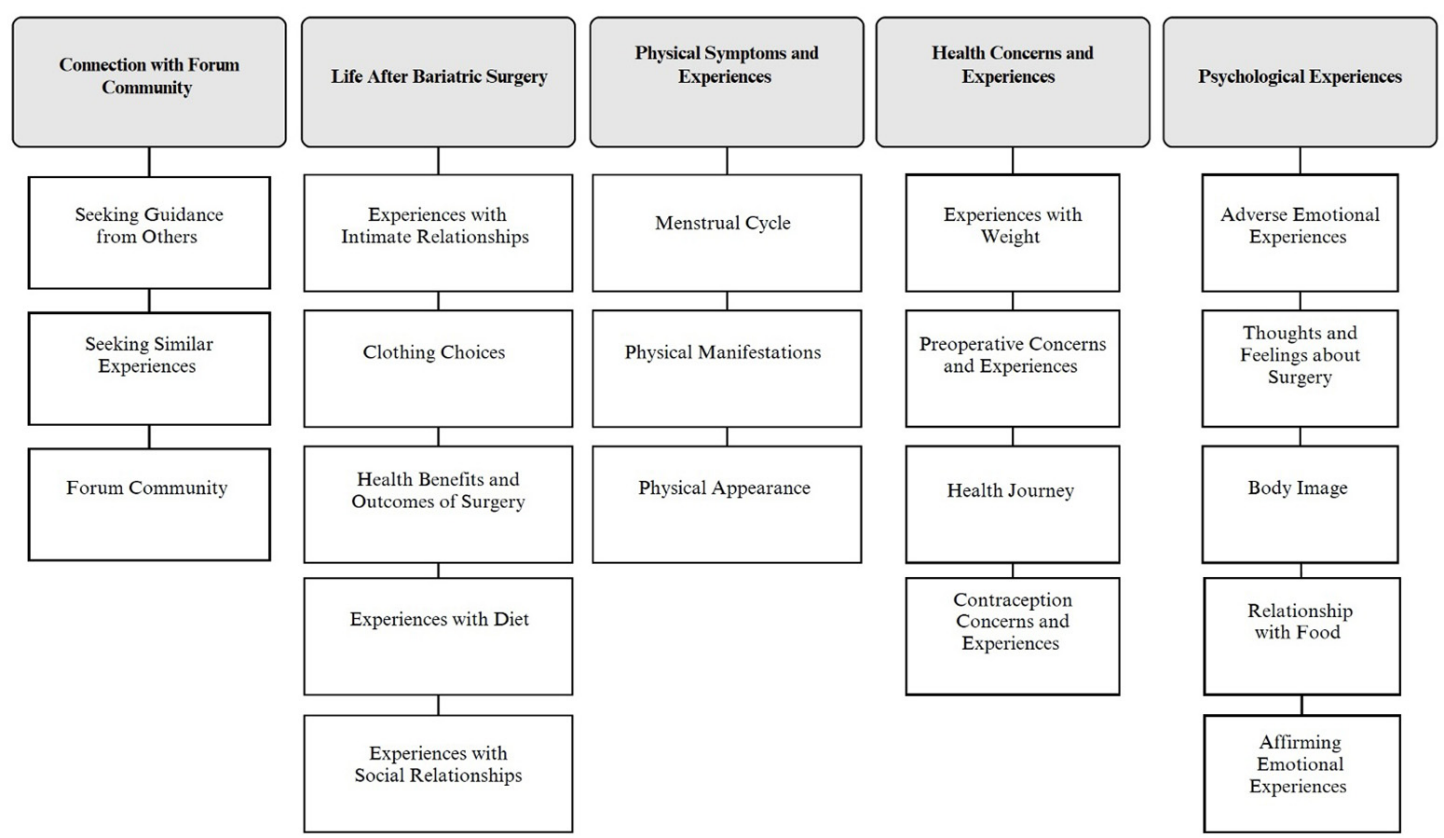
Overarching categories and associated subcategories.

From analysis of 289 posts, five categories were developed. Figure 2 illustrates the five categories and subsequent subcategories. The five categories and relevant subcategories are further summarised with illustrative quotations in Table 2. See Supplemental File 2 for full analysis structure with codes, subcategories and categories.

**Table 2.** Categories and subcategories with illustrative quotation(s).

*Category 1: Connection with forum community*	
Subcategory	Subcategory illustrative quotation(s)
Seeking guidance from others *n* = 128 (62.0%)	‘For those who have had surgery…if you look back, are there things that you wish you’d known about beforehand? I think I’m trying to find out as much as possible before having surgery and hoping I don’t go into information overload but use it wisely instead’. (Participant 97)
Seeking similar experiences *n* = 126 (61.0%)	‘So, does anyone have any good sports bra recommendations? I have started working out a lot and my regular VS [Victoria’s Secret] bras are no longer cutting it. I of course can go to VS [Victoria’s Secret] and get what they have but was kind of hoping for some other options. They have to be very supportive as I am a 38 DDD [Bra Size]’. (Participant 2)
Forum community *n* = 61 (29.0%)	‘It’s obvious that I’m still learning how to get familiar with this awesome little bariatric world! Most people (family and friends) I socialise with out here in the real world don’t really understand this whole bariatric lifestyle! So, I’m excited to be part of a virtual community who totally gets ME and YOU and You Too Girl! We are totally all in this together’. (Participant 165)
*Category 2: Life after bariatric surgery*	
Subcategory	Subcategory illustrative quotation(s)
Experiences with intimate relationships *n* = 79 (38.0%)	‘First, he said he is worried something will happen to me, then he says I can do it [weight loss] by exercise and diet, and then today he said that he met me heavy, and he wants me to be like how I was when we first met. Honestly, I think he is worried that if I get thin that I will leave him, which I would not leave him, but he told me that he will not support me on my decision to have it [bariatric surgery]. He said we will get a divorce’. (Participant 180). ‘I have just lost my first 50 lbs and I am starting to get a lot more confident in myself. I have started using the dating apps and now that the possibility of dating is more real, I am starting to get really freaked out’. (Participant 83)
Clothing choices *n* = 46 (22.0%)	‘Now that I am a size 10/12 and have lots of options, I find myself really enjoying fashion and style…I am starting to develop a kind of sexy professional style that is fun and funky - mixing up prints and pastels, wearing shorter length pants and different shape pants and lots of skirts!’ (Participant 41)
Health benefits and outcomes of surgery *n* = 44 (21.0%)	‘I am loving life again, and doing more things now that I could not do before I lost over 300 lbs. I don’t need to use a scooter or walker wheelchair to get around, I’m finally out of my bed, tying shoes by myself, getting dressed by myself, and many other things’. (Participant 84).
Experiences with diet *n* = 34 (16.0%)	‘I am just over two years out and I have gained 40 pounds back. After surgery I had no complications. Nothing made me sick, there was no food that I couldn’t eat or that I couldn’t tolerate. I did good for a little over a year then I hit a rough patch in life and went completely downhill. I was craving and eating sweets like it was nothing’. (Participant 143)
Experiences with social relationships *n* = 25 (12.0%)	‘Just today my sister is trying to give me advice to not feel nauseous. I just want to say to her you don’t know what I’m going through so shut the hell up. She’s like oh I talk to a friend of mine who had the surgery and she said this. It’s OK for us to go back to each other because we’ve all been through it but when other people start interjecting saying oh, I read this, or I saw that. I want to shoot them’. (Participant 53).
*Category 3: Physical symptoms and experiences*	
Subcategory	Subcategory illustrative quotation(s)
Menstrual cycle *n* = 136 (65.0%)	‘I’m too shy to talk to anyone about this. I have not had my period for over a year now and today I was surprised it came back. I heard that having bariatric surgery sometimes helps women get it. Does anyone know why? This may be TMI [Too Much Information] and if so sorry!’ (Participant 117).
Physical manifestations *n* = 44 (21.0%)	‘Did anyone else have vajayjay [vaginal] changes since surgery? I’ve always been a big water drinker which now isn’t so much but also feel like I’m having the beginnings of yeast infection or something just super dry feeling. If this makes any sense at all’. (Participant 17).
Physical appearance *n* = 33 (16.0%)	‘I’m 36 and almost 8 months out at 80 lbs down. I’ve been reading about loose skin from WLS [Weight Loss Surgery] and saw one source that said it takes about 2 years for your skin to go back to “normal”. While I’m grateful for my tool [Bariatric Surgery] and the weight loss, my wrinkled brown bag stomach looks sad and I’m not crazy about the turkey neck, either’. (Participant 32).
*Category 4: Health concerns and experiences*	
Subcategory	Subcategory illustrative quotation(s)
Experiences with weight *n* = 81 (39.0%)	‘I am new here and had the sleeve done on July 24, 2019. I started at 300 pounds and am now 223. I feel great but feel like I should have lost more. Is this normal?’ (Participant 130). ‘I had my gastric bypass in 2011 I had lost 120 pounds. I have gained 60 pounds back. I am totally disappointed in myself. I have tried everything to get it back off’ (Participant 142). ‘This morning the post op swelling in my abdomen has FINALLY taken a hike, but it appears that my boobs hitched a ride with it…that was an undesired side effect!’ (Participant 119).
Preoperative concerns and experiences *n* = 47 (23.0%)	‘I’ve been told that if I lose 17 lbs, which can realistically happen in 6 months, that I will no longer qualify [for bariatric surgery]. Which is causing some issues for me’ (Participant 73). ‘I’m so stuck thinking I should try and lose the weight naturally again (I have been overweight most of my life and dieted most of my life too). Or I should bite the bullet and go for surgery?’ (Participant 38).
Health journey *n* = 42 (20.0%)	‘On one hand, my surgeon is telling me that in his experience, PCOS [Polycystic Ovary Syndrome] goes away in women who successfully complete and keep the weight off after surgery. On the other hand, my OB-GYN [Obstetrician-Gynaecologist] is telling me she doesn’t think it will change anything other than I won’t have to take Metformin [medication] anymore… I’m so confused! I have no idea what to believe and I have never met anyone else in this situation before’. (Participant 123).
Contraception concerns and experiences *n* = 38 (18.0%)	‘I’d like to reach out to my gyno [Gynaecologist] and see about birth control options. I was on the pill before, but I am a little curious about the arm implant and the IUD [Intrauterine Device]. What has been everyone’s experience?’ (Participant 77). ‘I am seven days postop with the Sleeve. I have had an IUD [intrauterine device] for 8 months and thankfully because of the IUD I don’t have periods. However, 6 days postop I started to spot. Could this be related to the surgery? Had anyone experienced this?’ (Participant 67).
*Category 5: Psychological experiences*	
Subcategory	Subcategory illustrative quotation(s)
Adverse emotional experiences *n* = 56 (27.0%)	‘I’m 4 weeks post RNY [Roux-en-Y] operation. I’ve noticed that now that I’m unable to eat food I get angry about food. I get mad that I can’t eat, mad at my friends and family because they can eat what they want, and I even get angry at the fact that I drive past so many restaurants etc’. (Participant 94).
Thoughts and feelings about surgery *n* = 48 (23.0%)	‘Although I’m excited for this journey and ultimately the surgery, I’m a little apprehensive about it. Like it may not actually be a reality and I’m going to wake from this dream, and it’ll be psych! The jokes on me. My weight has been a struggle since puberty and I’m ready for that to end and start life as the woman I’ve always wanted to be’. (Participant 73).
Body image *n* = 35 (17.0%)	‘So, I am finishing the pre surgery process here in a couple weeks and it is starting to be real that I’m really going to get the surgery and life as I know it now is going to change. Has anyone dealt with fear of what their new body will look like? I’ve been plus size my entire life. I’m nervous as to what my new body will look like…Has anyone else felt this way?’ (Participant 203). ‘In an effort to embrace reality and with a healthy dose of encouragement from “Shrill” [TV Show], I decided I’m wearing a bikini this summer. Stretch marks, scars, loose skin, and all’ (Participant 74).
Relationship with food *n* = 27 (13.0%)	‘I’m on the second week of my puréed diet and I’ve really struggled with it. I keep eating food that’s not puréed and I know that it’s not what I’m supposed to be doing. I’m just hungry all the time and then I eat too much and hurt. I did some soul searching and realized that I can’t do this to myself, or I’ll ruin my chance of success…I’m realizing just how much food effects every facet of my life’. (Participant 94)
Affirming emotional experiences *n* = 14 (7.0%)	‘I feel great. I don’t feel like people are staring at me because of my size (not that they probably ever were). I don’t feel like I stick out like a sore thumb standing next to my thin family’ (Participant 34).

Counts and percentages were calculated based on the total number of participants. (*N* = 208).

### Connection with forum community

The most prominent category ‘Connection with Forum Community’ captured the experiences and concerns shared with the forum community. Women sought others’ medical, psychological, and social experiences after surgery as well as before undergoing the surgery themselves. Within this, women requested recommendations, such as what diet to follow or which clothing to wear. Further, there were general interactions that reflected a sense of community. Women shared general life experiences to seek support. Similarly, women sought a support network with others on similar surgical timelines. Others felt misunderstood by the wider community and experienced deeper understanding from the forum community.

### Life after bariatric surgery

In ‘Life After Bariatric Surgery’, experiences from different facets of life after bariatric surgery were shared. For example, concerns and experiences were raised within intimate relationships. On one hand, women were grateful for supportive partners. On the other hand, women experienced conflict or breakdown in intimate relationships, citing surgery as a contributing factor. Similarly, women were concerned about intimacy and dating, sharing sexual experiences with the online community for support and advice. Women shared general clothing advice with the community, and sought advice about issues with bras and/or underwear fitting after surgery and clothing goals (e.g. fitting into a smaller clothing size). Women discussed how their clothing options had expanded after weight loss and how their sense of style had shifted.

This category also encompassed women’s health and lifestyle outcomes after bariatric surgery. Many reported health and wellbeing benefits and shared this with the community. Specifically, improvements in the menstrual cycle, mobility, continence, and overall quality of life were reported. A common discussion topic among the community was ‘non-scale victories’, which were defined as positive health victories unrelated to numerical weight. Examples of these victories included being able to wear a wedding ring again, or no longer needing a seatbelt extender on an aeroplane. Regarding diet, challenges included dehydration and difficulty adhering to dietary guidelines, often leading to feelings of disappointment. Concerns about vitamin and mineral deficiencies after surgery were expressed, along with food cravings, sometimes during emotional distress. Socially, several women expressed frustration and concern about perceived stigma from others and worries about how others might view their decisions. They shared experiences of both supportive or unsupportive family regarding their surgery choice. Additionally, some were concerned about the impact their decision might have on their children, driven by a desire to set a healthy example.

### Physical symptoms and experiences

Within the ‘Physical Symptoms and Experiences’ category, women shared multiple experiences and concerns regarding physical symptoms or changes. Many sought advice on how bariatric surgery had affected the menstrual cycle, specifically whether others had encountered similar changes. They reported experiencing adverse menstrual symptoms, such as abnormal menstrual bleeding, Premenstrual Syndrome (PMS) symptoms and menstrual cycle pain. Most of these issues were unexpected, suggesting that many women were unaware of how the surgery could impact their menstrual cycle. A range of other adverse physical manifestations were reported, such as vaginal issues, gastrointestinal or bladder issues, nausea, dehydration, fatigue, unpleasant bodily odour, dry skin, unwell feeling and symptoms of dumping syndrome, where food moves from the stomach into the small bowel too quickly, inducing symptoms of nausea and vomiting (Ukleja, 2005). Women used the forum to seek advice and experiences with the issue in question. Regarding appearance, those in the postoperative phase often reported disdain towards loose skin. Likewise, women reported issues with sagging breasts after weight loss and concerns about the appearance of their hair after surgically induced hair thinning or loss. Women in the preoperative phase expressed worries about how their appearance might change after surgery.

### Health concerns and experiences

The ‘Health Concerns and Experiences’ category encompassed general discussions about health and wellbeing experiences. For example, women shared experiences with obesity related disease, such as Gastroesophageal Reflux Disease (GERD) or type 2 diabetes mellulitis. Mainly, women were concerned about amounts of weight loss and sought advice from the forum regarding weight loss stalls or struggles with weight loss after surgery. Similarly, women reported issues with weight regain following initial weight loss. Alternatively, concerns were raised about significant and undesired weight loss from breasts. Prior to surgery, women expressed personal goals for life after surgery, citing motivation to lose weight and feel healthier. Women expressed financial concerns regarding surgery, and general worries about surgery, including concerns about losing too much weight preoperatively to qualify. Women expressed experiences with the preoperative diet, struggling to cull foods or liquids such as sugar or caffeine that hold addictive characteristics. Lastly, women became frustrated with surgery delays, expressed uncertainty around telling others about their upcoming surgery, and were uncertain whether to undergo the surgery at all.

Women shared healthcare experiences unrelated to weight, including pregnancy, medication and other medical procedures. They discussed experiences and struggles with reproductive illness, like endometriosis, adenomyosis and Polycystic Ovary Syndrome (PCOS). Interactions with health professionals varied: one participant felt supported, while others faced conflicting medical opinions, leading to uncertainty. Women also raised questions about contraceptives, expressing concerns about weight gain and requested shared experiences with the contraceptive pill, IUD (Intrauterine Device), and the contraceptive patch, seeking advice on their use after surgery. Others shared adverse experiences with contraception, such as anxiety or depression and breakthrough bleeding.

### Psychological experiences

In the ‘Psychological Experiences’ category, a range of psychosocial issues and topics were discussed, with most psychological experiences captured being adverse. Women sought support by expressing that they felt worried or stressed, depressed, resentful, scared, upset or overwhelmed, and frustrated or angry. Some women felt alone, experienced weight-related embarrassment, and experienced other mental health challenges. Some women spoke unfavourably about themselves and expressed disappointment for failing to reach weight loss milestones. In this category, women expressed thoughts and feelings towards the surgery itself. Some women were excited and hopeful, however, fear of lifestyle change, nervousness for surgery and hesitance about surgery were also reported. After surgery, women experienced mixed feelings about weight loss and difficulty adjusting to a new stomach and lifestyle. Regarding body image, women reported insecurity and self-consciousness, especially when wearing revealing clothing or swimwear, and some women feared the bodily change associated with weight loss. This category included women’s psychological relationship with food, including if it was healthy or disordered and how it changed after surgery. Some experienced negative emotions towards food or alcohol, used food for comfort during times of emotional distress or displayed maladaptive eating behaviours. While some women regretted their surgery choice, others felt satisfied with their weight loss progress, were proud of their achievements, enjoyed rewarding themselves for reaching weight loss milestones and experienced improved self-esteem, and largely relished in sharing these positive experiences with the forum community.

## Discussion

This study provides an overview of the experiences and informational needs that women expressed in an online forum dedicated to bariatric surgery, with a focus on three aims. Firstly, we aimed to contribute knowledge regarding how and why women engaged in online peer support throughout the bariatric surgery process. Secondly, we explored the concerns and experiences that women shared on the forum. Thirdly, we sought to decipher ways in which preoperative education can be improved to better support women undergoing bariatric surgery. The key findings highlight the importance of online peer support forums, the common experiences shared to the forum and how these experiences can inform improvements in preoperative education and psychosocial support for those undergoing bariatric surgery.

### Engagement in online peer support

Regarding the first aim of this study, a key finding was the importance of online forum communities as a social support resource, consistent with previous research (Athanasiadis et al., 2022; Hossain et al., 2021; Koball et al., 2017). This study is the first qualitative study to focus on women’s experiences with multiple bariatric procedures using the Bariatric Pal forum. Within this forum, women offered emotional support offering empathy when sharing challenging experiences. Provision of informational support through sharing successful experiences and advice was evident, with women posting after surgery to compare experiences. This is consistent with previous studies examining online bariatric communities (Athanasiadis et al., 2022; Koball et al., 2017; Robinson et al., 2020; Scarano Pereira et al., 2022; Willmer and Salzmann-Erikson, 2018).

### The experiences and concerns shared to the forum

Concerning the second aim of this study, the present study provides valuable insights into what experiences and concerns women share with online peers regarding bariatric surgery. A key finding was that women shared experiences with the forum community to seek support and advice from peers that appeared unavailable from healthcare professionals regarding changes in reproductive health. Polycystic ovary syndrome (PCOS) and endometriosis were common discussion topics, and women often posted concerns about their menstrual cycle returning after surgery and weight loss, questioning if this was normal. However, it is known that anovulation, menstrual irregularity, and PCOS are common in women with obesity and that these issues usually improve after weight loss (Lee et al., 2020). Furthermore, the return of ovulation and improvement in menstrual regularity can justify the use of contraception. In the present study, women were concerned about whether contraceptive use was still appropriate after surgery and displayed uncertainty of the contraceptive options (e.g. Intrauterine Device (IUD)) available to them. This supports literature that contraception education is lacking after surgery (Mengesha et al., 2016; Riedinger et al., 2020),

Bariatric surgery is a life changing operation with impacts on mental wellbeing (Coulman et al., 2017). Most psychological experiences posted by participants were adverse. This study supports previous literature in that bariatric surgery candidates often have unrealistic expectations for weight loss (Homer et al., 2016). Consistently, women were disappointed with lack of weight loss during unrealistic timeframes. Concerns about weight regain were tainted with distress, disappointment and frustration. Body insecurity and self-consciousness were general themes and weight loss did not always alleviate insecurities, and instead, insecurities about excess skin were expressed, which has been observed in previous research (Altieri et al., 2017; Mento et al., 2022). This finding supports literature urging for body contouring cosmetic surgery to be discussed following bariatric surgery in obesity management (Altieri et al., 2017). Some women did embrace their bodies and took pride in weight loss achievements, however, these findings highlight the importance of ongoing psychological support to maintain healthy body image, eating habits and psychosocial wellbeing after bariatric surgery, as well as in the preparation phases.

### Implications for preoperative education

Pertaining to the third aim, the experiences women shared to the forum highlighted gaps in preoperative education. Preoperative education aims to manage unrealistic weight loss expectations and inform individuals about potential outcomes of surgery (Schlottmann et al., 2018). Despite this, candidates may not receive adequate information about the various physical, psychological, and social challenges associated with rapid weight loss and bariatric surgery (Wright et al., 2022). Moreover, when individuals feel ill-equipped with the education provided by the healthcare professionals they may turn to online support communities for advice and support. This was demonstrated in the present study with lack of knowledge evident in multiple areas. The experiences that women shared to the forum inform a need for enhanced preoperative education on the impact of rapid weight loss on reproductive health, menstrual health and fertility so that women feel equipped to manage these changes. Improved education from healthcare professionals surrounding contraceptive options needs to be considered to avoid unwanted pregnancy, for example, that is already unrecommended during rapid weight loss and subsequent hormonal changes (Riedinger et al., 2020).

Further, the findings regarding psychological wellbeing support a need for information, psychological and social support from health professionals, both in the preparation phases before surgery, and afterwards in order to address and manage candidate expectations of surgical outcomes and prevent distressing psychological experiences. Research surrounding psychosocial support from health professionals supports this, in that the provision of preoperative support that extends into the postoperative period may be beneficial in improving psychological well-being and weight loss maintenance (Van Zyl et al., 2024). Similar research suggests that individuals undergoing bariatric surgery prefer an integrative and stepped-care approach both pre- and postoperatively, with autonomy and access to future supports being considered important factors (Van Zyl et al., 2024). Therefore, the present study emphasises a need for enhanced, patient-centred and continuous psychosocial support, beginning from the preoperative phases, to enhance the postoperative outcomes of bariatric surgery.

### Strengths and limitations

A strength of using a qualitative methodology is that it enabled for rich accounts of consumer experiences to be explored, and the use of online forum raw and unfiltered data enabled for naturalistic account. Despite these strengths, findings should be interpreted with the acknowledgement of methodological limitations. There are demographic limitations, in that the majority of participants were from the United States of America (USA), a country with unique socio-political and economic conditions. Hence, experiences specific to USA individuals are overrepresented. Due to the public nature of online forum data, it was not possible to implement measures to control for participants’ baseline knowledge of surgical procedures, nor know the type of surgery participants had received unless it was explicitly stated in the content of the post. Further, this study was limited to cisgender women, and therefore further research could focus on cisgender men, or individuals with other gender identities, given the gender disparity and limited understanding of educational needs related to bariatric surgery within these groups.

In regards to the limitations of using online forum data, whilst forum posts are naturalistic accounts of real persons, not everyone can access the internet and this research cannot be applied to specific populations (Smedley and Coulson, 2021). Further, despite this study providing a comprehensive overview of the experiences and concerns shared to the forum, only opening posts from the forum were included for analysis: further analysis of complete threads may yield interesting insights. Also, due to the anonymity of the study, it was not possible to contact participants to seek clarification on post meanings. Future research could triangulate the results of this analysis with other qualitative methodological approaches such as interviews or surveys.

### Conclusion

This study highlighted unique considerations for women undergoing bariatric surgery and identified a gap in qualitative research on the gender-specific outcomes of surgery, considering whether preoperative education is adequate in addressing these. As this study was the first to qualitatively explore women’s experiences using the online forum Bariatric Pal, it provides insights into women’s use of online peer support networks and affirms the value of these networks as important information resources and sources of social support. Further research could explore how to enhance accessibility to online peer support networks and improve the structure of preoperative and postoperative care to better inform decision-making and enhance women’s experiences with bariatric surgery.

## Supplemental Material

sj-docx-1-hpq-10.1177_13591053251337218 – Supplemental material for The experiences and informational needs of women electing bariatric surgery: A qualitative content analysis of an online support forum Supplemental material, sj-docx-1-hpq-10.1177_13591053251337218 for The experiences and informational needs of women electing bariatric surgery: A qualitative content analysis of an online support forum by Jasmine Lester and Anna Chur-Hansen in Journal of Health Psychology

sj-docx-2-hpq-10.1177_13591053251337218 – Supplemental material for The experiences and informational needs of women electing bariatric surgery: A qualitative content analysis of an online support forum Supplemental material, sj-docx-2-hpq-10.1177_13591053251337218 for The experiences and informational needs of women electing bariatric surgery: A qualitative content analysis of an online support forum by Jasmine Lester and Anna Chur-Hansen in Journal of Health Psychology
