# Supplementary material for: The experiences and informational needs of women electing bariatric surgery: A qualitative content analysis of an online support forum
Source: J Health Psychol. 2025 May 8;30(14):4333–48. doi: 10.1177/13591053251337218 (PMC12678650; doi:10.1177/13591053251337218)
Supplement: sj-docx-1-hpq-10.1177_13591053251337218 – Supplemental material for The experiences and informational needs of women electing bariatric surgery: A qualitative content analysis of an online support forum [file sj-docx-1-hpq-10.1177_13591053251337218.docx]

**Supplementary File 2**

Categories, Subcategories, and Sub-Codes with Counts

| Category | Subcategory | Sub-Code | *n* | % of N |
| --- | --- | --- | --- | --- |
| Connection with Forum Community | Seeking Guidance from Others  *n* = 128 (62.0%) | Seeking advice from others after undergoing surgery  Seeking advice from others before undergoing surgery  Seeking medical advice  Asking if their experience is normal  Asking others what to expect  Asking for hope | 78  32  8  7  2  1 | 38.0%  15.0%  4.0%  3.0%  1.0%  0.5% |
|  | Seeking Similar Experiences  *n* = 126 (61.0%) | Seeking the medical experiences of others after surgery  Seeking the experiences of others before undergoing surgery  Seeking the psychological experiences of others after surgery  Seeking the social experiences of others after surgery  Seeking recommendations from others | 63  28  14  13  8 | 30.0%  13.0%  7.0%  6.0%  4.0% |
|  | Forum Community  *n* = 61 (29.0%) | Seeking a support network  Sharing life experiences  Spreading positivity to the community  Feeling understood by the community  Thankful to the community | 23  18  8  6  6 | 11.0%  9.0%  4.0%  3.0%  3.0% |
| Life After Bariatric Surgery | Experiences with Intimate Relationships  *n* = 79 (38.0%) | Impacts of surgery on intimate relationships  Conflict in intimate relationship  Dating after surgery  General sexual experiences  Concerns about relationship breakdown  Unsupportive partner  Concerns about intimacy  Supportive partner  Divorce after surgery  Experiencing low libido | 16  11  9  9  8  8  5  5  4  4 | 8.0%  5.0%  4.0%  4.0%  4.0%  4.0%  2.0%  2.0%  2.0%  2.0% |
| Clothing Choices  *n* = 46 (22.0%) | Questions about clothing  Experiences with clothing  Changes in clothing choices after weight loss  Issues with bras and underwear  Clothing goals  Sharing clothing advice | 12  11  9  8  3  3 | 6.0%  5.0%  4.0%  4.0%  1.0%  1.0% |  |
| Health Benefits and Outcomes of Surgery  *n* = 44 (21.0%) | Improvement in overall health and wellbeing  Non-scale victories  Improved quality of life  Improved mobility  Improved menstrual cycle  Improved continence | 19  12  6  4  2  1 | 9.0%  6.0%  3.0%  2.0%  1.0%  0.5% |  |
| Experiences with Diet  *n* = 34 (16.0%) | General eating habits and experiences  Diet after surgery  Issues with inadequate water consumption  Not adhering to diet guidelines  Experiencing food cravings  Concerns about vitamin and mineral deficiencies | 9  6  6  6  5  2 | 4.0%  3.0%  3.0%  3.0%  2.0%  1.0% |  |
| Experiences with Social Relationships  *n* = 25 (12.0%) | Perceived stigma from others  Worried about other’s opinions  Concerns about the impact of their surgery on their children  Supportive family  Unsupportive family | 9  7  5  2  2 | 4.0%  3.0%  2.0%  1.0%  1.0% |  |
| Physical Symptoms and Experiences | Menstrual Cycle  *n* = 136 (65.0%) | Impacts of surgery on the menstrual cycle  Concerns about the menstrual cycle changing after surgery  Experiencing abnormal menstrual bleeding  Experiencing Premenstrual Syndrome (PMS) Symptoms  Experiencing pain during menstrual cycle  Experiences with menopause | 52  28  18  16  13  9 | 25.0%  13.0%  9.0%  8.0%  6.0%  4.0% |
|  | Physical Manifestations  *n* = 44 (21.0%) | Experiencing discomfort or pain  Vaginal issues  Gastrointestinal Issues  Nausea  Dehydration  Bladder Issues  Fatigued  Symptoms of Dumping Syndrome  Unpleasant bodily odour  Dry Skin  Feeling unwell | 15  6  5  4  3  3  2  2  2  1  1 | 7.0%  3.0%  2.0%  2.0%  1.0%  1.0%  1.0%  1.0%  1.0%  0.5%  0.5% |
|  | Physical Appearance  *n* = 33 (16.0%) | Issues with loose skin  Concerns about hair  Issues with sagging breasts  Concerns about how appearance will change after surgery | 16  8  5  4 | 8.0%  4.0%  2.0%  2.0% |
| Health Concerns and Experiences | Experiences with Weight  *n* = 81 (39.0%) | Experiences with obesity-related disease  Concerns about amount of weight loss  Experiencing significant weight loss in breasts  Concerns about weight loss stalls  Experiencing weight regain  Struggling with weight loss  Long term struggle with weight  Impacts of weight on daily life  Concerns about stretching stomach | 17  12  12  11  8  7  5  5  4 | 8.0%  6.0%  6.0%  5.0%  4.0%  3.0%  2.0%  2.0%  2.0% |
|  | Preoperative Concerns and Experiences  *n* = 47 (23.0%) | Goals for life after surgery  Concerns about qualifying for surgery  Experiences with preoperative diet  General concerns about the surgical process  Unsure about surgery  Unsure how to tell others about surgery  Frustrated at surgery delays  Cannot afford surgery | 9  8  8  8  5  4  3  2 | 4.0%  4.0%  4.0%  4.0%  2.0%  2.0%  1.0%  1.0% |
|  | Health Journey  *n* = 42 (20.0%) | Experiences with Polycystic Ovary Syndrome (PCOS)  Experiences with medication  Experiences with other medical procedures  Interactions with medical professionals  Experiences with endometriosis and adenomyosis  Experiences with pregnancy  Experiencing strange dreams  Impacts of COVID-19 Pandemic | 13  7  7  6  5  2  1  1 | 6.0%  3.0%  3.0%  3.0%  2.0%  1.0%  0.5%  0.5% |
|  | Contraception Concerns and Experiences  *n* = 38 (18.0%) | Questions about contraception  Concerns about contraception causing weight gain  Experiences with the contraceptive pill  Experiences with IUD’s  Adverse experiences with contraception  Experiences with other forms of contraception | 12  7  7  5  4  3 | 6.0%  3.0%  3.0%  2.0%  2.0%  1.0% |
| Psychological Experiences | Adverse Emotional Experiences  *n* = 56 (27.0%) | Frustrated or angry  Upset or overwhelmed  Disappointed in self  Scared  Feeling alone  Resentful  Unfavourable self-talk  Worried or stressed  Depressed  Experiencing mental health issues  Embarrassment | 9  7  6  6  5  5  5  5  4  3  1 | 4.0%  3.0%  3.0%  3.0%  2.0%  2.0%  2.0%  2.0%  2.0%  1.0%  0.5% |
|  | Thoughts and Feelings about Surgery  *n* = 48 (23.0%) | Excited and hopeful for surgery journey  Difficulty adjusting after surgery  Pleased with decision to have surgery  Nervous for surgery  Experiencing mixed feelings about weight loss  Hesitant about surgery  Regretful of decision to have surgery  Fear of lifestyle changes | 17  8  6  6  3  3  3  2 | 8.0%  4.0%  3.0%  3.0%  1.0%  1.0%  1.0%  1.0% |
|  | Body Image  *n* = 35 (17.0%) | Insecure about body  Distorted body image  Fear of new body  Feeling self-conscious about body  Increased body confidence after surgery | 15  7  5  5  3 | 7.0%  3.0%  2.0%  2.0%  1.0% |
|  | Relationship with Food  *n* = 27 (13.0%)  Affirming Emotional Experiences  *n* = 14 (7.0%) | Maladaptive eating behaviours  Food as a comfort  Adverse emotions towards food and alcohol  Pleased with weight loss progress  Proud of self  Feeling more secure in self  Rewarding self after weight loss | 15  7  5  7  3  2  2 | 7.0%  3.0%  2.0%  3.0%  1.0%  1.0%  1.0% |
|  |  |  |  |  |

*Note.* Counts were calculated based on the number of participants that posted under each code. Percentages were calculated using the total number of participants (N = 208).
